# Exploring the mechanism of luteolin by regulating microglia polarization based on network pharmacology and in vitro experiments

**DOI:** 10.1038/s41598-023-41101-9

**Published:** 2023-08-23

**Authors:** Tianyue Wang, Yuanjun Yin, Xinyu Jiang, Yanmin Ruan, Jiawen Xu, Xiaowei Hu, Tianyi Li, Lisheng Chu, Lin Li

**Affiliations:** 1 Department of Physiology, Zhejiang Chinese Medical University, Hangzhou, 310053 China; 2 The Second Clinical Medical College, Zhejiang Chinese Medical University, Hangzhou, 310053 China; 3 The First Clinical Medical College, Zhejiang Chinese Medical University, Hangzhou, 310053 China

**Keywords:** Cell polarity, Computational biology and bioinformatics, Pharmacology

## Abstract

Neuroinflammation manifests following injury to the central nervous system (CNS) and M1/M2 polarization of microglia is closely associated with the development of this neuroinflammation. In this study, multiple databases were used to collect targets regarding luteolin and microglia polarization. After obtaining a common target, a protein–protein interaction (PPI) network was created and further analysis was performed to obtain the core network. Molecular docking of the core network with luteolin after gene enrichment analysis. In vitro experiments were used to examine the polarization of microglia and the expression of related target proteins. A total of 77 common targets were obtained, and the core network obtained by further analysis contained 38 proteins. GO and KEGG analyses revealed that luteolin affects microglia polarization in regulation of inflammatory response as well as the interleukin (IL)-17 and tumor necrosis factor (TNF) signaling pathways. Through in vitro experiments, we confirmed that the use of luteolin reduced the expression of inducible nitric oxide synthase (iNOS), IL-6, TNF-α, p-NFκBIA (p-IκB-α), p-NFκB p65, and MMP9, while upregulating the expression of Arg-1 and IL-10. This study reveals various potential mechanisms by which luteolin induces M2 polarization in microglia to inhibit the neuroinflammatory response.

## Introduction

Injuries to the CNS trigger neuroinflammation which can result in the emergence of neurodegenerative illnesses^1^ including Alzheimer disease (AD), Parkinson disease (PD), amyotrophic lateral sclerosis (ALS), and multiple sclerosis (MS)^2^. Inflammatory responses are generated by glial cells under the influence of infection, mechanical injury, toxic metabolites, and autoimmunity^3^. Neuroinflammation is a complex immune response in neural tissues which eliminates pathogens, clears cellular debris and inhibits the spread of infection in its early stages. However, prolonged and excessive inflammatory response damages in the CNS can both lead to and aggravate neurodegenerative diseases^4^. For the treatment of neurodegenerative diseases, reducing the inflammatory response of the CNS is considered to be a potential point of entry^5^.

Microglia are a highly specialized type of macrophages found in the thin-walled tissues of the CNS^6^ which are involved in the CNS inflammatory response^7^. In their resting state, microglia are involved in the development of the CNS and the maintenance of homeostasis. Pathological factors stimulate microglia to convert them into an activated state where they assume an amoeboid morphology^8^. Interestingly, recent studies indicate that the activation state of microglia has both M1 and M2 phenotypes^9–11^. When it comes to the M1 phenotype, microglia with iNOS frequently display pro-inflammatory traits and emit pro-inflammatory substances including TNF-, IL-6, and IL-1^12^. Microglia that have polarized to the M2 phenotype exhibit proteins like Arg-1 and CD206 and produce anti-inflammatory substances like IL-10 and TGF-β1^13,14^. Studies in recent years have also identified an inflammatory protease, known as MMP9^15^. This enzyme plays a major role in regulating inflammation and can also be used for disease course prognostication and predicting the risk of disability^16^. Current experimental studies have shown a relationship between MMP9 and microglia polarization but these experiments are limited in number and scope^17,18^.

There remains enormous value in investigating this relationship further. Microglia polarization is often identified via specific markers of different phenotypes. Among the many approaches put in place to combat inflammation in the CNS, regulating the activated microglia conversion to the M2 phenotype may be a better way to suppress the CNS inflammatory response^19^.

Currently many drugs are reported to inhibit the onset of microglia activation, and only a small number of compounds have been shown to modulate the polarization state of microglia^20^. Therefore the exploration of such drugs is of profound significance^21^.

Luteolin, a flavonoid, is present in a variety of plants, including flowers, herbs, vegetables, and spices^22^. Previous research results have demonstrated that luteolin has good anti-inflammatory^23^, anti-cancer^24^, and antioxidant properties^25^. Excitingly, luteolin has demonstrated neuroprotective effects that can be used to treat a range of neurological illnesses such as AD^26,27^, PD^28,29^ and traumatic brain injury (TBI)^30,31^. The compound can also easily penetrate the blood–brain barrier (BBB), decreasing the cytotoxic effects of oxidative stress and free radicals at the site of injury which limits the inflammatory response after CNS injury^32^.

**Figure 1:**
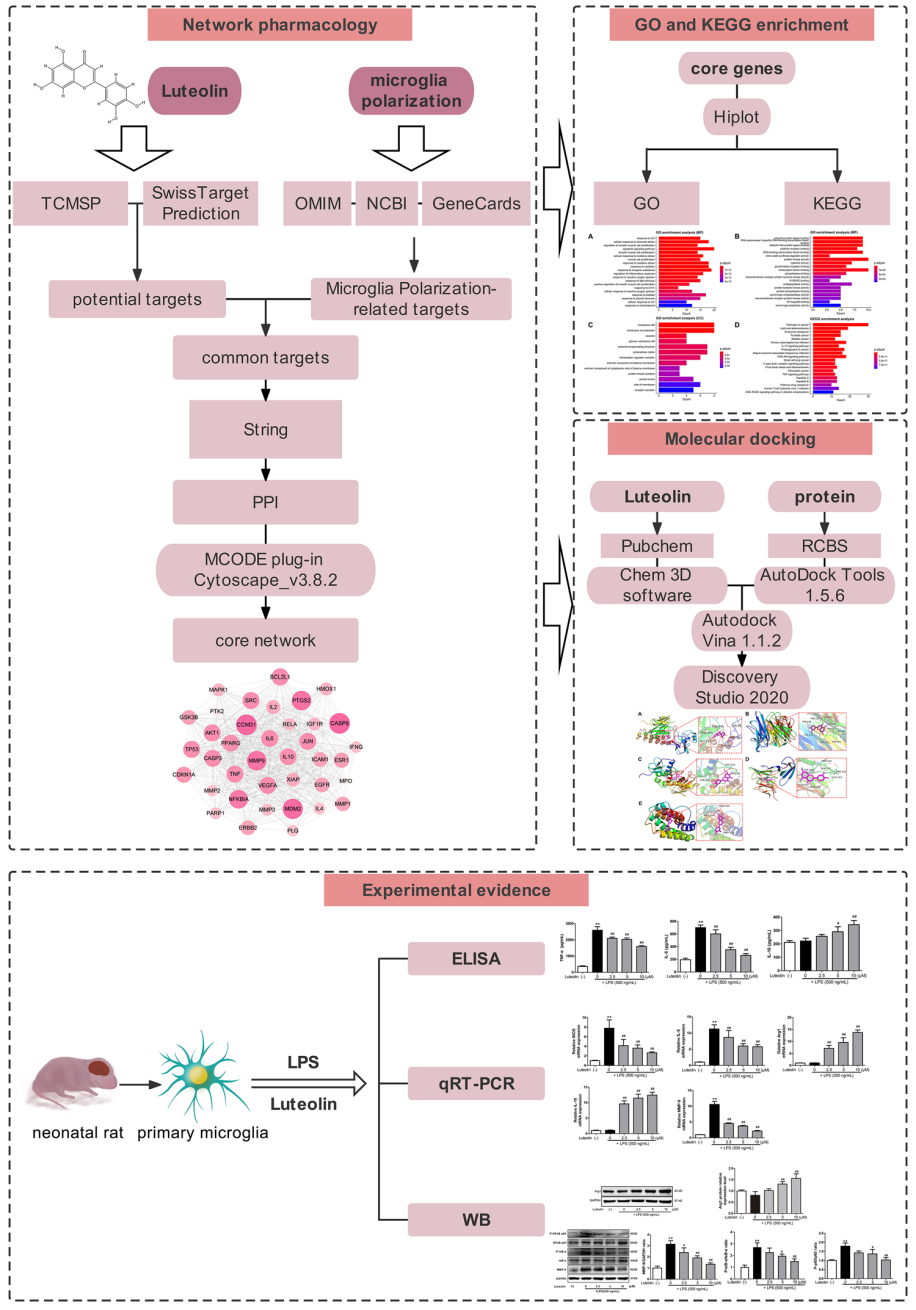
Flow chart of the research work.

Although the neuroprotective effect of luteolin is well established^33^, it is not clear if it regulates the polarization of microglia as a part of its anti-inflammatory activity. In our study, we use two methods in combination to investigate the pharmacodynamic properties of luteolin in a comprehensive and multifaceted manner. First, network pharmacology, which is a new method that can be used to determine mechanism of action of active ingredients in traditional Chinese medicine through pharmacokinetic evaluation^34,35^, was used to evaluate the ability of luteolin to affect microglia polarization. We then performed in vitro cellular experiments using primary microglia to demonstrate the ability of the compound to regulate cell polarization and suppress inflammatory reactions. In addition to providing in vitro verification of network pharmacology results, this combined method also provides reference values for future clinical treatments regarding inflammation of the CNS. The detailed workflow diagram of the study is presented in Fig. 1.

## Materials and methods

### Luteolin and microglia polarization related target genes prediction and intersection analysis

The TCMSP database (http://tcmspw.com/tcmsp.php) and SwissTargetPrediction database (http://www.swisstargetprediction.ch/) were used to predict the potential targets of luteolin, and Uniprot (https://www.UniProt.org/) was utilized to translate the targets into gene names. Microglia polarization-related targets were collected through the OMIM (https://www.omim.org/), NCBI (https://www.ncbi.nlm.nih.gov/) and GeneCards databases (https://www.genecards.org/), by searching with the keyword "microglia polarization". Import drug and disease target data into the online Venn diagram production website (http://www.bioinformatics.com.cn/) for intersection analysis and export Venn diagram data files and common targets for further study.

### Construction of a PPI network

PPI is an important part of the network of various biochemical reactions in the organism. We selected the STRING database (https://cn.string-db.org/) for online analysis of the common targets obtained from the above intersection, selected the species "Homo sapiens", derived the protein interaction network, and exported it as a "tsv "file, to build a visual PPI network map in Cytoscape_v3.8.2.

### Acquisition of a core network and prediction of key targets

MCODE plugin is a clustering algorithm that builds functional modules based on the relationship of edges and nodes in a large network of actions to find key sub-networks and genes for easy downstream analysis. The core network in PPI is analyzed using the MCODE plug-in to derive the highest scoring network for further study. In addition, hub nodes are identified using CytoHubba. The important genes in the core network may be determined and ordered according to their scores using the CytoHubba plug-in and the MCC algorithm, offering some guidance for following studies.

### GO and KEGG enrichment analyses

The GO database contains Biological Process (BP), Cellular Component (CC) and Molecular Function (MF), which are used to reveal the potential mechanism of action of the target in the organism. The KEGG database integrates large-scale data to analyze genetic information and enrich for potential signaling pathways^36^. Hiplot is a comprehensive data computing and visualization cloud platform based on the R language. clusterProfiler package in R is used to annotate and visualize relevant KEGG passages and GO terms. The clusterProfiler package produced GO and KEGG enrichment analyses in Hiplot. We relied on the Hiplot online website (https://hiplot.com.cn/) for GO and KEGG enrichment analyses of the core network, with the p-threshold set at 0.05. A total of 20 enrichment entries were then selected for further in-depth study of signaling pathways and biological functions related to microglia polarization. Later, a correlation visualization pathway and biological function-target connection map were created using Cytoscape v3.8.2.

### Molecular docking

A theoretical simulation technique called molecular docking is used to analyze intermolecular interactions and forecast intermolecular binding affinities and patterns. Molecular docking was carried out with Autodock Vina to investigate the interactions between luteolin and the proteins in the core network. First, the luteolin 3D molecular structure was retrieved from Pubchem (https://pubchem.ncbi.nlm.nih.gov/). Chem 3D software was used to perform the energy minimization calculation and output the 3D structure, which was saved as a mol2 format file. Then, 38 protein crystal structures were retrieved from the RCBS Protein Data Bank (https://www.rcsb.org), dehydrated, hydrogenated, and calibrated for Gasteiger charge using AutoDock Tools 1.5.6, and saved as pdbqt files. Next, molecular docking anchoring calculations were performed using Autodock Vina 1.1.2 to determine the docking conformation between the protein and the chemical component with the lowest binding energy, which can yield the degree of interaction of luteolin with the target. Finally, the target proteins (IL-6, IL-10, MMP9, NF-BIA, and TNF) that were also monitored in this experiment were visualized and analyzed using Discovery Studio 2020.

### Animal

Primary microglia cells were extracted from the cortexes of postnatal day 1 to day 3 Sprague Dawley rats obtained from the Sino-British SIPPR/BK Laboratory Animal Center (Shanghai, China). All animal experiments were performed in accordance with the ARRIVE guidelines. The animal study was reviewed and approved by Institutional Animal Care and Use Committee of the Laboratory Animal Research Center of Zhejiang Chinese Medical University (reference number: IACUC-20220328-23). The authors confirm that all methods were carried out in accordance with relevant guidelines and regulations.

### Materials

Luteolin (CAS: 491-70-3, MW: 286.24, purity ≥ 96%) was purchased from Shanghai Yuanye Biotechnology Co. Ltd., (Shanghai, China). Fetal bovine serum (FBS) (Cat No: 10099-141C) was from Gibco BRL (Grand Island, United States). Dulbecco’s Modified Eagle Medium (Cat No: C11995500BT) was obtained from ThermoFisher Biochemical Products Co. Ltd., (Beijing, China). Cell Counting Kit-8 (CCK-8) (Cat No: GK10001) was from GlpBio Technology (Montclair, USA). NO assay kit (Cat No: S0021) was from Shanghai Beyotime Biotechnology Co. Ltd. (Shanghai, China). Rat TNF-α ELISA kit (Cat No: EK0526), rat IL-6 ELISA Kit (Cat No: EK0412), and rat IL-10 ELISA Kit (Cat No: EK0418) were from Boster Biological Technology co. ltd., (Wuhan, China). Arg1 (Cat No: 16001-1-AP) was from Proteintech (Rosemont, IL, USA). MMP-9 (Cat No: AB19016) was from Millipore (Billerica, MA, USA), NFκBIA (IκB-α, Cat No: sc-1643), p-IκB-α (Cat No: sc-8404), p-NFκB p65 (Cat No: sc-136548), NFκB p65 (Cat No: sc-8008), GAPDH (Cat No: sc-47724) was from Santa Cruz Biotechnology (Dallas, TX, USA). Poly-d-lysine-coated (PDL) (Cat No: P6407) and lipopolysaccharide (LPS) were from Sigma-Aldrich (San Louis, MO, USA). Ionized calcium binding adaptor molecule 1 (Iba1) antibody (Cat No: 019-19741) was from WAKO (Osaka, Japan). RNAiso Plus (Cat No: 9109), PrimeScript™ RT Master Mix (Cat No: RR036A) and TB Green^®^ Premix Ex Taq™ (Cat No: RR420A) Kit were from TaKaRa (Tokyo, Japan).

### Primary microglia cell culture

As previously mentioned, primary microglia cell culture was carried out^37,38^. In brief, after removing meninges, the cortical tissues from neonatal Sprague Dawley (SD) rat (P0–P3) were sliced into 1 mm^3^ blocks and transferred to an ice-cold tissue homogenizer with 3 mL ice-cold Dulbecco's modified eagle medium (DMEM). Homogenize the tissue in a glass tissue grinder on ice. Using 30 strokes, then passed through a 70-μm nylon mesh cell strainer and plated on 0.01% PDL 75 cm^2^ culture flasks supplement with 10% heat-inactivated fetal bovine serum (FBS), 100 U/mL penicillin and 100 μg/mL streptomycin. The cell culture medium was refreshed 24 h after initial preparation and every 3–4 days subsequently. For 10–14 days, DMEM was used to support these mixed glial cells. Microglia cells were then separated from the mixed glia by shock. The purity of microglia was assessed by immunofluorescent staining using an anti-Iba1 antibody, and only samples with more than 95% purity were used for the study.

### Immunocytochemistry

Primary rat microglial cells (1 × 10^4^ per well) were plated in 96-well plates for 24 h. Cells were then fixed with 4% paraformaldehyde for 15 min, and permeabilized with 0.3% Triton X-100 for 10 min. Then cells were blocked with 5% bovine serum albumin (BSA) for 1 h. Subsequently, the cells were incubated with an anti-Iba1 (1:100 dilution) antibody overnight at 4 °C, followed by incubation with the corresponding secondary antibody at 37 °C for 1 h in the dark. After 3 washes with PBS, cells were stained with 4,6-diamino-2-phenyl indole (DAPI) at room temperature for 5 min. Fluorescent microscopy (DMIL, Leica Microsystems, Germany) was used to get the images. The number of cells that stained positively in three randomly selected fields for each specimen was tallied for analysis.

### Cell counting kit-8 (CCK-8) assay

Cell viability was assessed using the CCK-8 assay in line with the manufacturer's recommendations. Primary rat microglial cells (1 × 10^4^ per well) were plated in 96-well plates and cultured overnight. Five different concentrations of luteolin (1 μM, 2.5 μM, 5 μM, 10 μM and 20 μM) or LPS (lipopolysaccharide) (500 ng/mL) were treated with cells. As a vehicle control, cells were treated with DMSO (0.1%, v/v). 10 μL of CCK-8 regent was added to each well after 24 h of incubation, and the plate was then incubated at 37 °C for another 4 h. Using a microplate reader (TECAN Infinite 200 pro, Austria), optical absorbance was determined at 450 nm.

### Griess assay

To determine how much stable nitrite was created in the culture medium using the nitrate reduction technique, nitric oxide (NO) generation was photometrically measured. Primary rat microglial cells (1 × 10^4^ per well) were plated in 96-well plates, and pretreated with 2.5, 5 or 10 μM luteolin for 2 h, followed by LPS (500 ng/mL) treatment for 24 h. The process was also completed using the Griess reagent according to the instructions for the NO assay kit. A microplate reader (TECAN Infinite 200 pro, Austria) was used to detect optical absorbance at 540 nm. Using a standard solution of sodium nitrite, NO concentrations were estimated.

### ELISA

Primary rat microglial cells (5 × 10^4^ per well) were seeded in 24-well plates, and pretreated with 2.5, 5 or 10 μM luteolin for 2 h, followed by LPS (500 ng/mL) treatment for 24 h. After the cultured media were collected, the levels of TNF-α, IL-6 and IL-10 were measured by using the corresponding rat ELISA kits. The optical absorbance was evaluated at 450 nm by using a microplate reader (TECAN Infinite 200 pro, Austria).

### qRT-PCR

Primary rat microglial cells (4 × 10^5^ per well) were seeded in 6-well plates, and pretreated with 2.5, 5 or 10 μM luteolin for 2 h, followed by LPS (500 ng/mL) treatment for 12 h. Following the manufacturer's directions, total RNA was extracted from primary rat microglial cells using RNAiso Plus. PrimeScript™ RT Master Mix was used to reverse-transcribe total RNA (500 ng) into cDNA according to the protocol provided with the reagent. Quantitative PCR was performed using TB Green^®^ Premix Ex Taq™ on the CFX96 Real-Time System instrument (BIO-BAD, USA), using the *Gapdh* gene as the internal reference. The sequence of PCR primers is shown in Table 1.

**Table 1:** Primers used for qRT-PCR.

Target genes	Forward primer sequence (5′–3′)	Reverse primer sequence (5′–3′)
iNOS	CTCCTTCAAAGAGGCAAAAATA	CACTTCCTCCAGGATGTTGT
IL-6	GAGGATACCACTCCCAACAGACC	AAGTGCATCATCGTTGTTCATACA
Arg-1	TCCTTAGAGATTATCGGAGCG	GTCTTTGGCAGATATGCAGG
IL-10	TGCCTTCAGTCAAGTGAAGAC	AAACTCATTCATGGCCTTGTA
MMP-9	GACCAGGATAAGCTGTATGG	CGGCACTGAAGAATGATCTA
GAPDH	GCCAAGGCTGTGGGCAAGGT	TCTCCAGGCGGCACGTCAGA

### Western blot analysis

Primary rat microglial cells (6 × 10^5^ per well) were seeded in 6-well plates, and pretreated with 2.5, 5 or 10 μM luteolin for 2 h, followed by LPS (500 ng/mL) treatment for 24 h. RIPA lysis buffer (including a 1% protease inhibitor cocktail) was used to extract the total protein from primary rat microglial cells. In accordance with the recommendations of the manufacturer, the bicinchoninic acid assay (BCA) was used to determine the protein concentration. 10% sodium dodecyl sulfate polyacrylamide gel (SDS-PAGE) was used to separate the protein samples, and the membranes were made of polyvinylidenefluoride (PVDF). After blocking with 5% (v/v) non-fat milk for 2 h, membranes were incubated with primary antibodies including anti-Arg1 (1:1000 dilution), anti-MMP-9 (1:1000 dilution), anti-p-NFκB p65 (1:500 dilution), anti-NFκB p65 (1:500 dilution), anti-p-IκB-α (1:500 dilution), anti-IκB-α (1:500 dilution) and anti-GAPDH (1:1000 dilution) overnight at 4 °C. After washing, the membranes were incubated with a horseradish peroxidase-conjugated secondary antibody (1:5000 dilution) at 37 °C for 2 h. An improved chemiluminescence detection kit was used to see protein bands. The ChemiDoc™ Imaging System (BIO-BAD, USA) was used to visualize the Western blot results, and ImageJ software was used to assess the band density. Information about antibodies can be seen in Table 2.

**Table 2:** Antibody-related information.

Antibodies name	Reagent specifications	Cat no.	Batch no.	Producers
Iba1	50 μg	019-19741	LEP3218	WAKO
Arg-1	150 μL	16001-1-AP	109643	Proteintech
p-NF-κB	100 μL	sc-136548	C2621	Santa Cruz Biotechnology
NF-κB	100 μL	sc-8008	C2421	Santa Cruz Biotechnology
p-IκBα	100 μL	sc-8404	C1021	Santa Cruz Biotechnology
IκBα	100 μL	sc-1643	B1221	Santa Cruz Biotechnology
MMP-9	1.0 mg/mL	AB19016	3218964	Millipore
GAPDH	1 mL	sc-32233	L0617	Santa Cruz Biotechnology

### Statistical analysis

The results were expressed as mean ± standard deviation (SD) and analyzed by one-way analysis of variance (ANOVA) followed by Tukey’s post hoc test with SPSS 20.0 software. Values of p < 0.05 indicated statistically significant differences.

### Ethics statement

All animal experiments were performed in accordance with the ARRIVE guidelines. The animal study was reviewed and approved by Institutional Animal Care and Use Committee of the Laboratory Animal Research Center of Zhejiang Chinese Medical University (reference number: IACUC-20220328-23). The authors confirm that all methods were carried out in accordance with relevant guidelines and regulations.

## Results

### Identifying luteolin and microglia polarization-related targets and common targets based on databases

**Figure 2:**
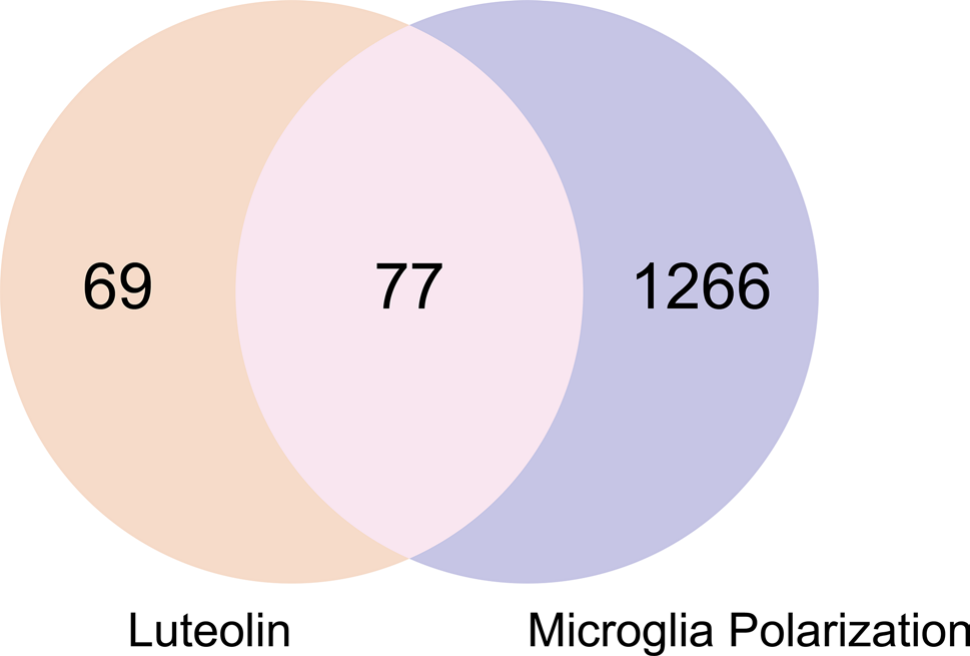
Venn diagram. The figure shows that luteolin and microglia polarization have a total of 77 intersecting targets that can be used as potential targets for drug action.

After collecting and summarizing the information from multiple databases and removing the redundant information, a total of 146 potential targets related to luteolin (Supplementary Table 1) and 1434 targets corresponding to microglia polarization (Supplementary Table 2) were obtained. According to the Venn diagram (Fig. 2), 77 common targets of luteolin and microglia polarization were obtained after intersection analysis (Supplementary Table 3).

### PPI network graph

**Figure 3:**
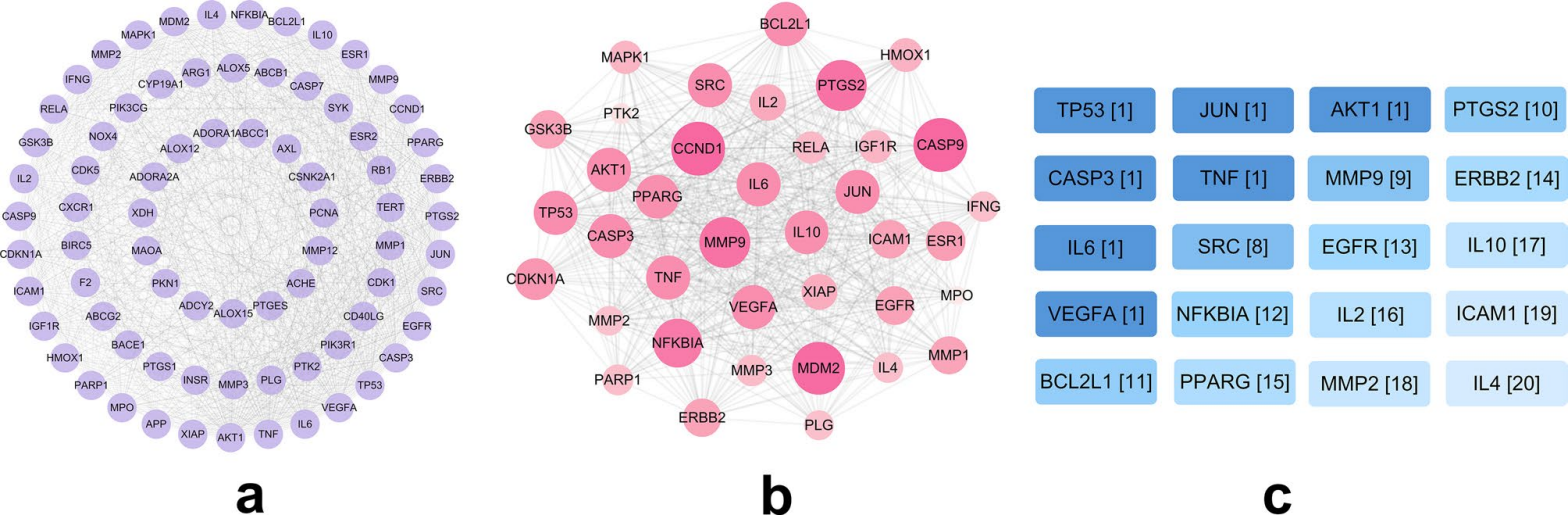
PPI graph and core network. (**a**) PPI relationship network, the edge corresponding to each node represents its degree of correlation with other nodes in the network. (**b**) Core network, the darker the color and larger the shape of the node, the more important the node is in the core network. (**c**) Key nodes in the core network. The nodes are labeled with the corresponding target names and MCC algorithm score ranking. The darker the color, the more critical the node is in the core network.

A PPI relational network of common targets was constructed based on the default algorithm of the STRING database. The network contained 76 nodes and 1042 edges with an average node degree of 27.1. Next, the tsv data file was transformed into a visual PPI network graph using Cytoscape_v3.8.2 (Fig. 3a).

### Constructing a core network and obtaining key genes

First, the PPI relationship network was clustered and analyzed using the MCODE plug-in, and the top-ranked sub-network with a score of 31.081 was selected, which contained 38 nodes and 575 edges (Fig. 3b). Then, the hub nodes in the core network were identified by the CytoHubba plug-in and its MCC algorithm, and ranked based on their scores (Fig. 3c). The higher the score, the more critical the node in the core network. Finally, it was observed that the scores of study-related targets such as MMP9, IL-6 and TNF were ranked higher and available for further study.

### Results of GO and KEGG enrichment analyses

**Figure 4:**
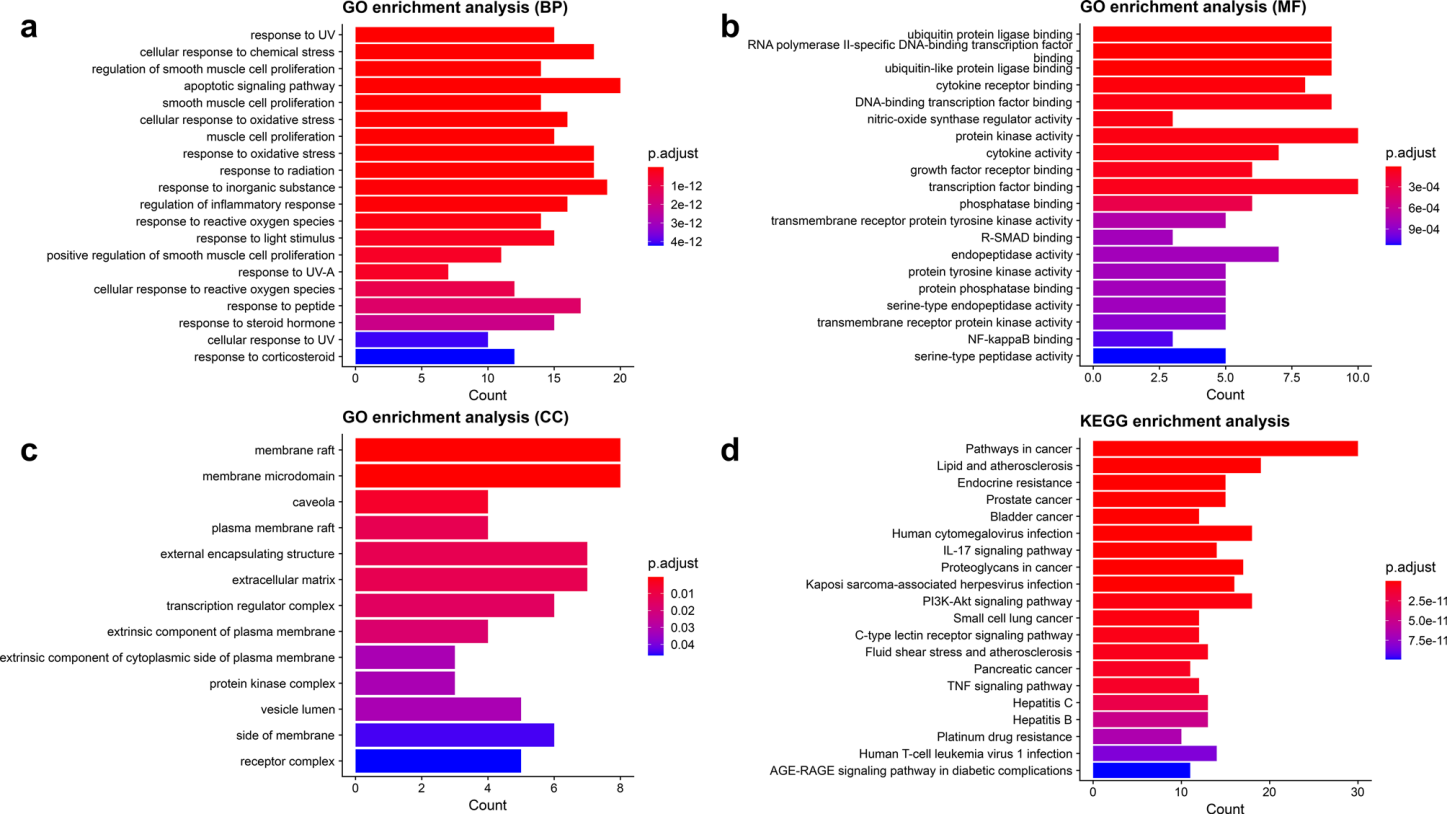
Histogram of the top 20 GO and KEGG enrichment terms. Terms of BP (**a**), MF (**b**), CC (**c**) and KEGG signaling pathway (**d**) are shown. In the bar graphs, the smaller the p-value and the longer the bars, the higher the enrichment.

**Figure 5:**
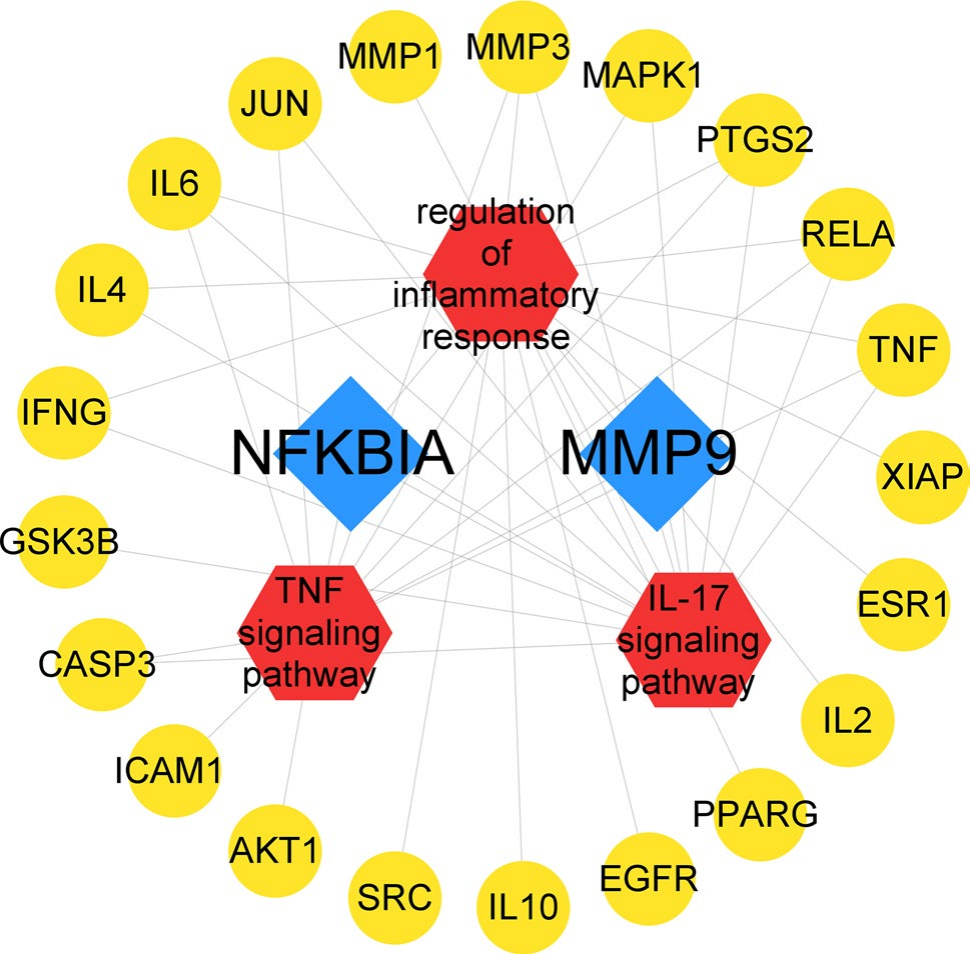
Signaling pathway and biological process-target relationship diagram. The red V-shaped nodes represent the corresponding signaling pathways and biological processes, the yellow circular nodes and blue diamond nodes represent the targets associated with each pathway, where the blue prismatic nodes represent NFKBIA and MMP9, which are present in all three pathways.

In order to better understand the different ways that luteolin affects microglia polarization at the systemic level, enrichment analyses using GO and KEGG were conducted. Based on the parameters set in the study conditions, the results of enriched MF, CC, BP and KEGG signaling pathways were obtained (Fig. 4). Based on the p-values and predetermined relevance to this experiment, we selected the IL-17 signaling pathway, the TNF signaling pathway, cellular response to oxidative stress, response to oxidative stress, and regulation of inflammatory response for further analyses. By importing these pathways and related genes into Cytoscape_v3.8.2 to construct a visual linkage map (Fig. 5), we found that MMP9 was included in all the five pathways with certain influence, which provided some guiding directions for our subsequent experiment.

### Molecular docking analysis

**Figure 6:**
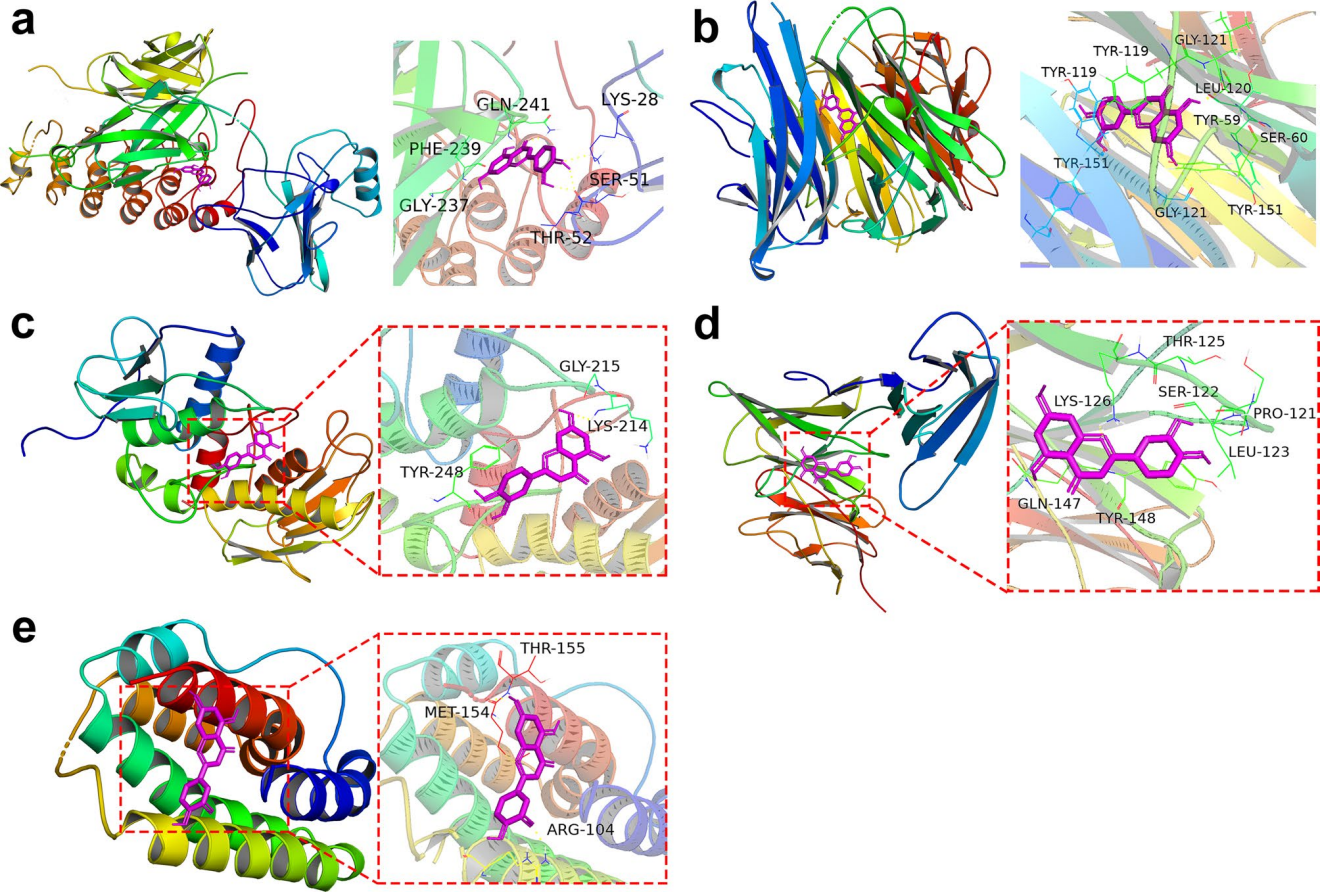
Molecular docking models of luteolin with NFKBIA (**a**), TNF (**b**), MMP9 (**c**), IL-6 (**d**) and IL-10 (**e**).

The molecular docking was completed using AutoDock Vina software, and the lowest binding energies of IL-6, IL-10, MMP9, NFκBIA and TNF to luteolin were − 6.8 kcal/mol, − 6.4 kcal/mol, − 7 kcal/mol and − 7.9 kcal/mol, respectively, showing good binding effects. As shown in Fig. 6, the best docking mode for luteolin and the target protein. The results of molecular docking of luteolin with 38 proteins can be seen in Table 3.

**Table 3:** The affinity test results of luteolin with 38 proteins.

Protein	PDB ID	Affinity (kcal/mol)
ERBB2	3PP0	− 10.3
MMP3	1HY7	− 9.8
PARP1	5WS1	− 9.7
MPO	4C1M	− 9.5
PTGS2	5F19	− 9.3
GSK3B	1Q41	− 9.3
ESR1	1UOM	− 9
RELA	3QXY	− 9
EGFR	4I24	− 9
JUN	2G01	− 8.9
MAPK1	1PME	− 8.8
SRC	2BDF	− 8.7
MMP2	3AYU	− 8.6
AKT1	4GV1	− 8.2
NFKBIA	1IKN	− 8.2
PLG	5UGG	− 8.2
IFNG	1FYH	− 8
HMOX1	3CZY	− 8
TNF	2AZ5	− 7.9
MMP1	1CGE	− 7.9
PPARG	6T9C	− 7.9
IGF1R	1JQH	− 7.8
CASP3	4PS0	− 7.8
CDKN1A	5E0U	− 7.7
CASP9	1JXQ	− 7.4
PTK2	2ETM	− 7.3
MDM2	4OGN	− 7.2
MMP9	4JIJ	− 7
IL6	1N26	− 6.8
XIAP	3HL5	− 6.7
BCL2L1	1MAZ	− 6.6
TP53	2J21	− 6.6
IL2	1M48	− 6.5
IL10	1LK3	− 6.4
CCND1	2W96	− 6.3
IL4	2B8U	− 6.1
VEGFA	1MKK	− 5.9
ICAM1	5MZA	− 5.1

### Luteolin suppressed pro-inflammatory mediator production and promoted anti-inflammatory M2 markers expression in LPS-stimulated primary microglia cells

**Figure 7:**
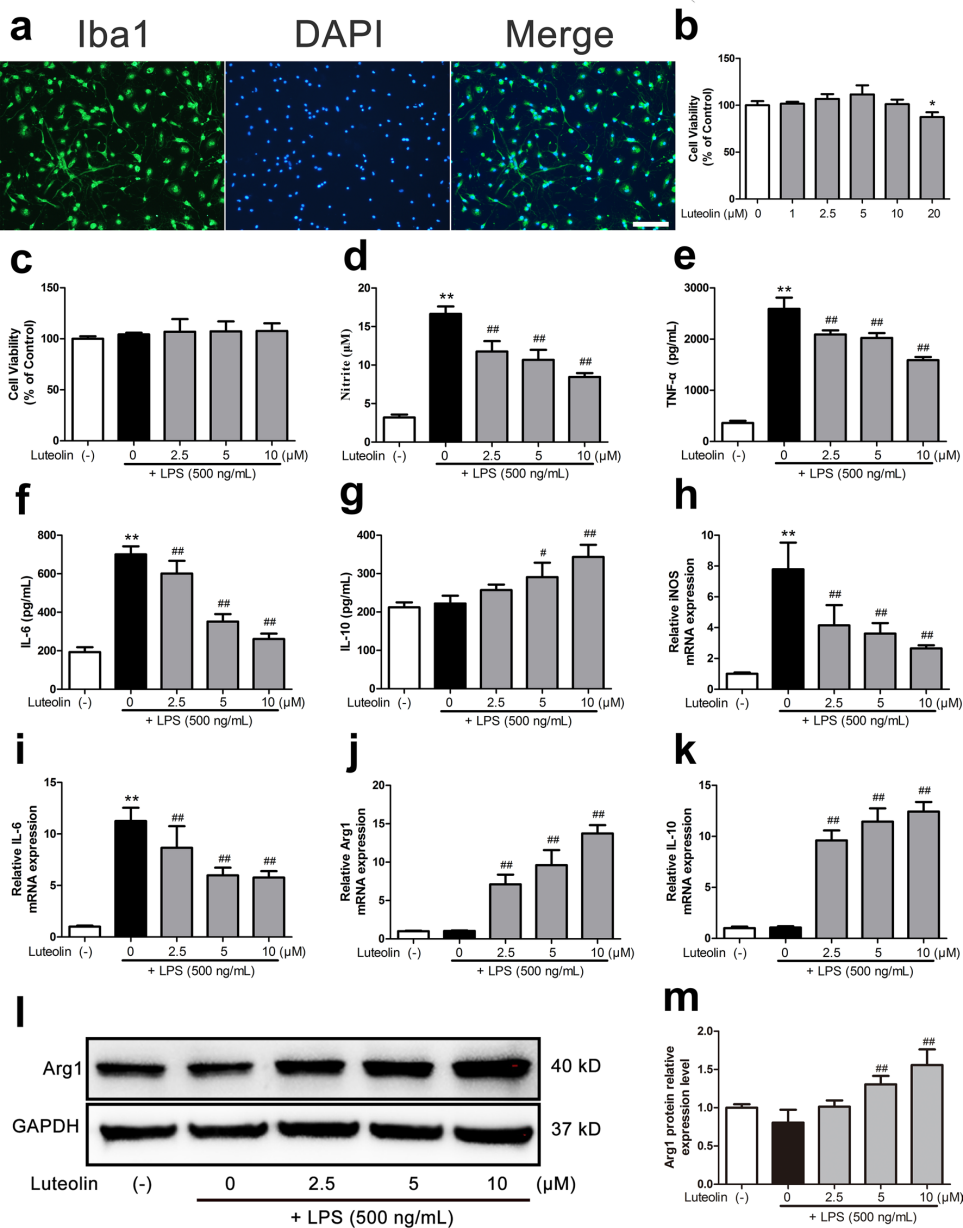
Luteolin suppressed pro-inflammatory mediators production and promoted anti-inflammatory M2 markers expression in LPS-stimulated primary microglia cells. (**a**) Identification of primary microglia cells. Immunostaining using an antibody targeting Iba1 (green), which is a marker for microglia cells and DAPI (blue) stain for cell nucleus. Scale bars: 100 µm. (**b**) Primary microglia cells were incubated with various concentrations of luteolin (1, 2.5, 5, 10, 20 μM) for 24 h to investigate the cytotoxicity. (**c**–**g**) Primary microglia cells were pretreated with the indicated concentrations of luteolin for 2 h followed by stimulation with 500 ng/mL LPS for another 24 h or (**h**–**k**) 12 h. (**c**) CCK8 assays. (**d**–**g**) The media were collected and the concentrations of NO, TNF-α, IL-6 and IL-10 were determined using the Griess reagent or ELISA kits. (**h**,**i**) Luteolin suppressed the iNOS and IL-6 mRNA expression, (**j**,**k**) promoted the mRNA expression of M2 microglial markers (Arg1 and IL-10) in LPS-stimulated primary microglia cells as determined by qRT-PCR. (**l**,**m**) The protein expression of Arg1 in primary microglia cells by western blot assay. *p < 0.05, **p < 0.01 compared with the control group, ^#^*p* < 0.05, ^##^*p* < 0.01 compared with the LPS group (**a**,**h**–**m**, n = 3, **b**–**g**, n = 5).

Due to the similarities between their phenotypic and in vivo condition, primary microglia cells, which have more complicated biological properties than other cell types, are frequently used in neuroinflammatory research^39^. Iba1 immunofluorescence histochemistry was used to identify the purity of primary microglia. The result found that the purity was above 98%, indicating that the culture method was feasible and subsequent experiments could be carried out (Fig. 7a).

The therapeutic effects of luteolin in primary microglia cells were then confirmed. In order to determine the cytotoxicity of luteolin on primary microglia cells, we first subjected rat primary microglia cells through a CCK-8 assay. After treatment with different concentrations of luteolin (1 μM, 2.5 μM, 5 μM, 10 μM and 20 μM) for 24 h, we determined that the safe concentration range of luteolin was ≤ 10 μM (Fig. 7b). Thus, luteolin at the doses of 2.5 μM, 5 μM, 10 μM were used in subsequent experiments.

Next, we explored at luteolin's ability to prevent LPS from activating microglia. LPS, which makes up the majority of gram-negative bacteria's outer membrane, has been demonstrated to significantly raise the production of pro-inflammatory cytokines in vitro and to activate microglia cells^40^. Gradient concentrations of luteolin cotreatment for 24 h did not appreciably change this LPS-stimulated viability improvement (Fig. 7c).

As determined by Griess assays and ELISA, luteolin (2.5 μM, 5 μM, 10 μM) significantly inhibited LPS-induced NO production (*p* < 0.01), pro-inflammatory cytokines (TNF-α and IL-6) (*p* < 0.01) and remarkably promoted anti-inflammatory cytokine (IL-10) (*p* < 0.05 or *p* < 0.01) secretion in the culture media of primary microglia cells (Fig. 7d–g). These findings were found to be dose-dependent in nature.

Most importantly, it was discovered that M1 and M2 polarizations of microglia could be recognized by their unique markers. As shown in Fig. 7h–k, the results of qRT-PCR showed that luteolin treatment significantly reduced the mRNA levels of M1 surface markers (iNOS) (*p* < 0.01) and pro-inflammatory cytokines (IL-6) (*p* < 0.01), and remarkably increased the mRNA levels of M2 surface markers (Arg-1) (*p* < 0.01) and anti-inflammatory cytokines (IL-10) (*p* < 0.01). Next, The LPS stimulation caused decrease in the protein expression of M2 associated markers Arg1, were altered in luteolin treated primary microglia cells (*p* < 0.01) (Fig. 7l,m). According to the aforementioned findings, luteolin suppresses the production of pro-inflammatory M1 mediators while encouraging the development of anti-inflammatory M2 indicators in primary microglia cells that have been activated by LPS.

### Expression of core target and related proteins

**Figure 8:**
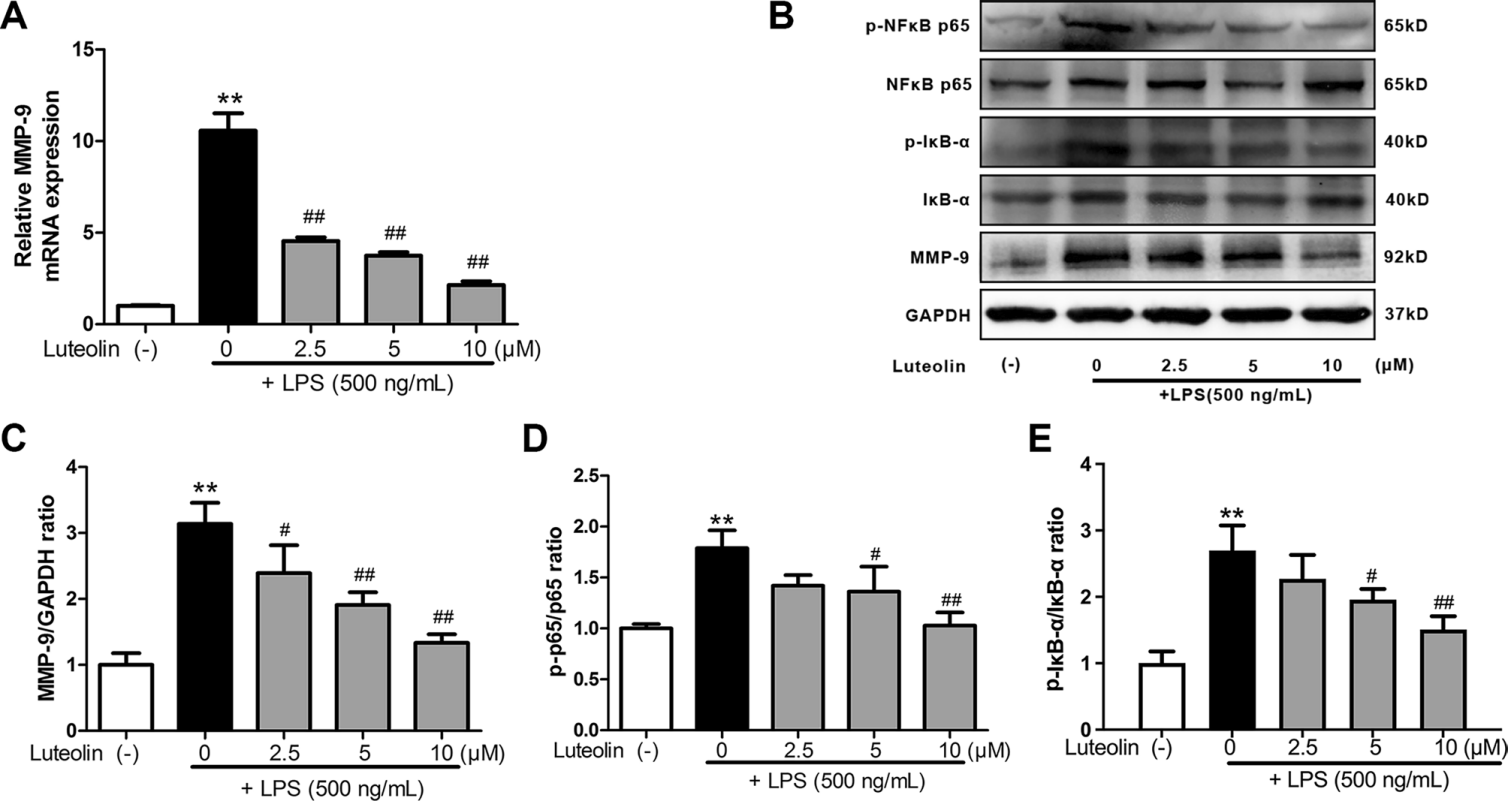
(**A**) The MMP-9 mRNA expression determined by qRT-PCR. (**B**) Representative images from Western blots for the proteins. (**C**) MMP-9/GAPDH ratio. (**D**) p-p65/p65 ratio. (**E**) p-IκB-α/IκB-α ratio. *p < 0.05, **p < 0.01 compared with the control group, ^#^*p* < 0.05, ^##^*p* < 0.01 compared with the LPS group (n = 3).

qRT-PCR and western blot analyses revealed that the LPS group had significantly greater levels of MMP-9 expression than the control group did. The luteolin-treated group showed significantly decreased MMP-9 expression in a concentration-dependent manner when compared with the LPS group (*p* < 0.01) (Fig. 8A–C). As shown, the expression level of p-NFκB p65/NFκB p65, p-IκB-α/IκB-α in the LPS group were significantly higher than when compared with the control group (*p* < 0.01), while the luteolin group markedly down-regulated compared with the LPS group (*p* < 0.05 or *p* < 0.01) (Fig. 8B,D,E).

## Discussion

In recent years, there has been increased focus on the relationship between microglia M1/M2 polarization and neuroinflammation^41^. Notably, this inflammatory response can disrupt the BBB causing neurodegenerative diseases^42^. Accordingly, we can infer that controlling the M1/M2 polarization of microglia could provide a new way to reduce the inflammatory response, thus decreasing the incidence of neurodegenerative diseases. The work in this study provides a framework to generate pharmaceutical interventions that regulate microglia polarization directly. This foundation will ultimately be used clinically to prevent the downstream development of neurodegenerative disease.

The use of network pharmacology assists in the discovery of biologic targets and mechanisms of action that are suitable for targeting by constructing a series of network relationship maps and conducting gene enrichment analyses^43^. Regarding luteolin, studies thus far have only demonstrated that it inhibits microglia activation and reduces cognitive impairment^33^. Microglial polarization and the mechanisms involved remain less well studied. This study, however, advances our knowledge base by demonstrating the feasibility of using network pharmacology followed by experimental validation to show that luteolin itself affects microglia polarization.

In a correlative analysis of network pharmacology, we used multiple databases to gather as much information as possible on luteolin and the targets related to microglia polarization, yielding a total of 77 common targets. For the obtained common targets, we used the MCODE plugin in Cytoscape_v3.8.2 to extract a total of 38 targets from the core network for subsequent analysis. Further narrowing of the subjects of analysis can allow some weakly interacting proteins to be eliminated, resulting in a core network with tighter protein interactions for follow-up GO and KEGG enrichment analyses^44^. After our enrichment analyses of the above genes, we found that such genes are closely related to the occurrence of inflammatory signaling pathways (IL-17 and TNF signaling pathway) and inflammation regulation, laterally responding to the potential therapeutic mechanism of luteolin. We also conducted molecular docking of luteolin with the corresponding core proteins, in which binding energies below − 5 kcal/mol suggest tight binding between small-molecule reagents and proteins^45^. The results in Table 3 show that luteolin can efficiently bind to all key proteins, which demonstrates the feasibility of luteolin for affecting microglia polarization. We also presented the visual analysis of the molecular docking of the selected proteins involved in this cellular experiment in this way in Fig. 6.

**Figure 9:**
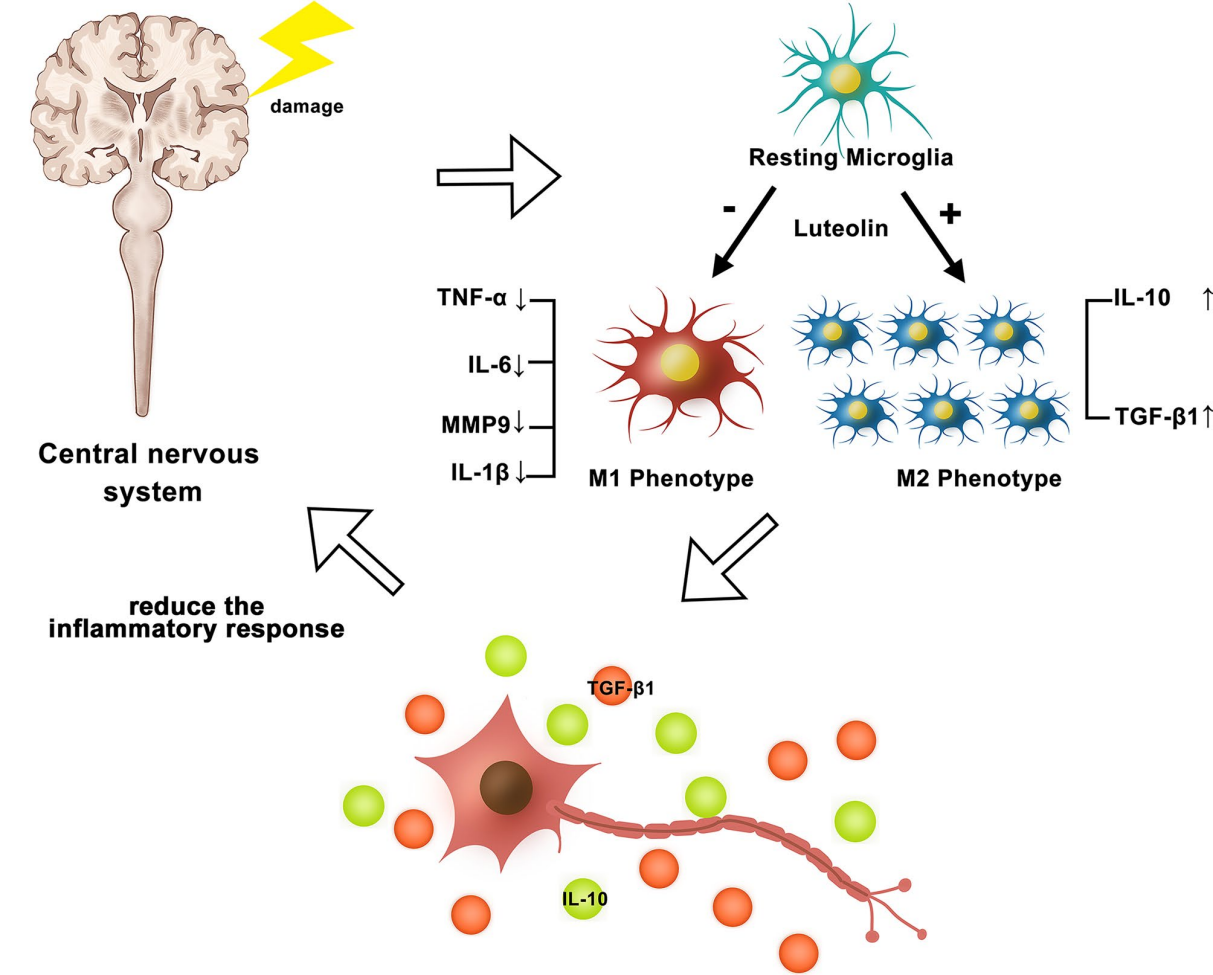
Mechanisms related to microglia polarization. Microglia can differentiate from resting state to M1 or M2 type upon stimulation. Luteolin induces microglia differentiation to M2 to promote the release of anti-inflammatory factors, reduce the inflammatory response and protect the CNS.

Through in vitro testing, the preliminary outcomes of network pharmacology and molecular docking investigations were then confirmed. In previous studies, we have found that microglia do not only exhibit resting and activated forms, but have two active phenotypes, the M1 and M2 types^46^. The M2 type controls the immune response to suppress the inflammatory response, whereas activation of the M1 type causes the production of pro-inflammatory substances that aggravate the inflammatory response in the body^19^. The mechanisms involved in microglia polarization can be seen in Fig. 9. In clinical practice, we strive for a balance between pro- and anti-inflammatory responses, as a sustained strong pro-inflammatory response can be damaging to the body^47^. Based on the study of the microglia activation state, we chose primary microglia in our in vitro cell experiments. Compared to traditional cell lines, primary microglia simulate in vivo biochemical reactions more faithfully, and the relevant experimental results obtained will be more reliable^37,38^. LPS is recognized for controlling microglia's M1-type polarization and facilitating the release of pro-inflammatory proteins, both of which support neuroinflammatory responses^48,49^. We cultured LPS-stimulated microglia with luteolin and identified that the expression of iNOS was downregulated, while the content of Arg-1 showed an upward trend (Fig. 7). These findings show that microglia can polarize to the M2 phenotype in the presence of luteolin, indicating that luteolin can, to some extent, influence microglia polarization, given that iNOS and Arg-1 are markers of M1 and M2 microglia, respectively. Therefore, we believe that the increased Arg1 expression is due to Luteolin promoting LPS-induced conversion of M1-type microglia to M2-type rather than increasing the number of microglia. Next, we performed ELISA and qRT-PCR on the relevant factors analyzed from the network pharmacology. The results exhibited that after exposure to luteolin, the expression of proinflammatory markers including IL-6 and TNF-α decreased while that of IL-10, a key anti-inflammatory mediator, increased^50^. Ultimately, we have demonstrated that luteolin has strong anti-inflammatory effects.

Current studies on microglia polarization have found that the CNS reduces inflammatory response and regulates microglia polarization by controlling the oxidative stress response after injury^51,52^. Among them IL-17 and TNF signaling pathways play a crucial role, which is also reflected in the results of our KEGG enrichment analysis. The proinflammatory IL-17 signaling pathway involves the synthesis of pro-inflammatory cytokines, chemokines, and, specifically for our findings, matrix metalloproteinases, which upregulates the expression of inflammatory genes^53^. The activation of the atypical NF-κB pathway, namely the TNF signaling pathway, induces apoptosis, thereby affecting cell survival, differentiation, proliferation, and migration^54,55^.

Interestingly, NFκBIA, also known as IκB-α, is involved in both of these pathways, and IκB-α is closely related to the protein NFκB^56^. After reviewing the relevant reference, we can clearly find that IκB-α is in the upstream position of the two signaling pathways, and its presence can well inhibit NFκB from entering the nucleus and alleviate the inflammatory response^57,58^. When IκB-α is phosphorylated, its inhibitory ability is greatly reduced, allowing NFκB activity to rise into the nucleus and the expression of related inflammatory factors to begin^59,60^. The expression of MMP9 rises when NFκB enters the nucleus. Previous experimental studies have also found that upregulation of MMP9 enables microglia activation and that reducing MMP9 expression inhibits its activation, protects the integrity of the BBB and attenuates the inflammatory response^61–63^. Our experimental results indeed show that luteolin can reduce the expression of IκB-α and NFκB as a way to inhibit the production of MMP9 in microglia.

After an in-depth analysis of the biochemical reactions and signaling pathways, we were led to explore the relationship between MMP9 and microglia polarization. MMP9, which is widely expressed in humans as a matrix metalloproteinase, can affect a variety of biological processes and multiple signaling pathways as well as plays an important role in PPI^64^. It is also involved in the physiological processes of neurogenesis, glial cell production, and brain plasticity, but is strictly regulated because it can be harmful when it is active^65,66^. At the time of neuronal injury and neuroinflammation, MMP9 exerts a major effect in regulating inflammation. Microglia promote the release of cytokines and free radicals after the massive production of MMP9, which in turn causes breakdown of the vascular basal lamina^67^, further degrading the proteins of the extracellular matrix, increasing the permeability of the BBB. The breakdown of BBB subsequently exacerbates post-ischemic endothelial damage, leading to cell death which allows microglia to migrate more smoothly^68–71^. According to a similar study, luteolin can reduce the size of cerebral infarction in rats following cerebral ischemia via inhibiting MMP9 expression^72^. However, there is no relevant experimental study on the inhibition of MMP9 expression by luteolin to promote M2 polarization in microglia.

We found through our WB experiments that the expression of MMP9, p-NFκB p65/NFκB p65 and p-IκB-α/IκB-α in microglia was definitively reduced after the addition of luteolin. Accordingly, luteolin not only inhibits the inflammatory response but also has a good potential in regulating a number of mechanisms of microglia polarization. Specifically, we have demonstrated the ability of luteolin to affect the polarization of microglia to the M2 type and the likely mechanism of this action via affecting IκB-α, NFκB and MMP9 in both IL-17 and TNF signaling pathways. These processes can be seen in Fig. 8.

However, our study has some limitations. One limitation is that the accuracy of the information contained in the analyzed databases was not verified, which may have affected our conclusion. In addition, although we have initially verified the relevant effects of luteolin, we need to carry out subsequent experiments in animal models to investigate the relevant mechanisms in more depth and to provide more effective reference values for clinical treatment.

## Conclusion

In the present research, we assessed luteolin's potential capacity to influence microglia's polarization toward the M2 phenotype and made predictions about the corresponding mechanisms of action based on network pharmacology. Then we experimentally demonstrated the effects of luteolin in modulating microglia polarization direction and attenuating inflammatory response.

### Supplementary Information

Supplementary Information.

## Data Availability

Data is contained within the article or supplementary material.
