# Supplementary material for: Exploring the mechanism of luteolin by regulating microglia polarization based on network pharmacology and in vitro experiments
Source: Sci Rep. 2023 Aug 23;13:13767. doi: 10.1038/s41598-023-41101-9 (PMC10447507; doi:10.1038/s41598-023-41101-9)
Supplement: Supplementary file 1 — Supplementary Information. [file 41598_2023_41101_MOESM1_ESM.pdf]

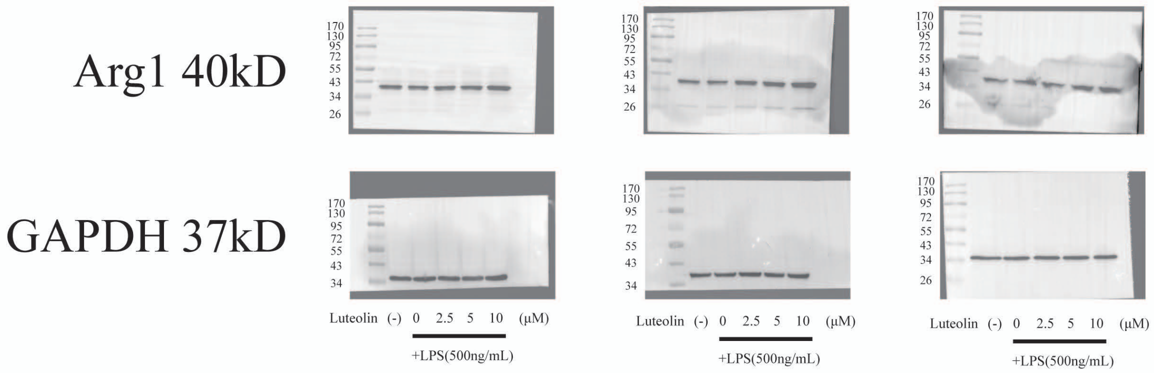


Supplement Figure 1. The original immunoblot images of Arg1 and GAPDH. The experiment was independently repeated three times.

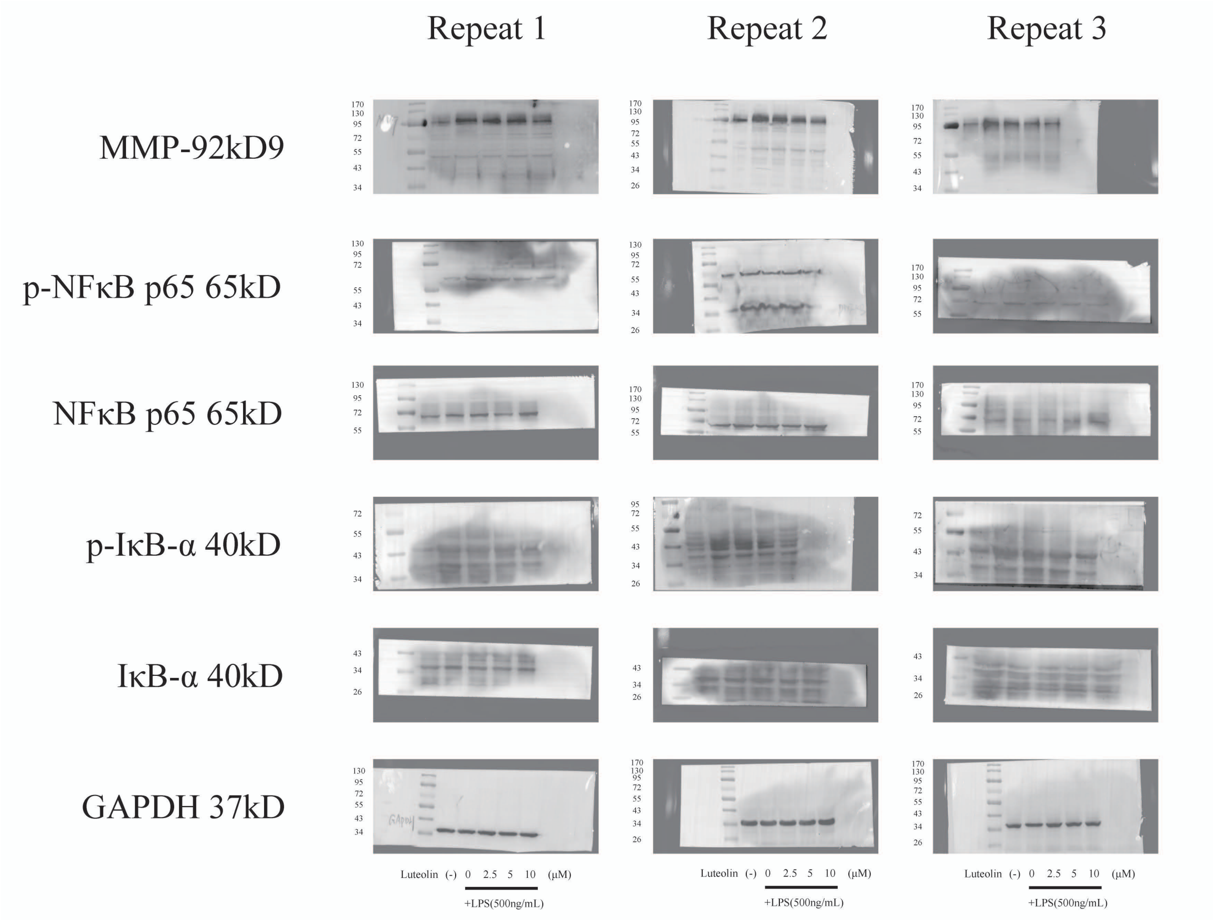


Supplement Figure 2. The original immunoblot images of MMP-9, p-NFkB, NFkB, p-IκB-α, IκB-α and GAPDH. The experiment was independently repeated three times.

Supplementary table 1. Potential targets for Luteolin

| **target name** |
|---|
| PTGS1 |
| AR |
| PTGS2 |
| PRSS1 |
| NCOA2 |
| DPP4 |
| PIK3CG |
| RELA |
| EGFR |
| AKT1 |
| VEGFA |
| CCND1 |
| BCL2L1 |
| CDKN1A |
| CASP9 |
| MMP2 |
| MMP9 |
| MAPK1 |
| IL10 |
| RB1 |
| CDK4 |
| TNF |
| JUN |
| IL6 |
| CASP3 |
| TP53 |
| NFKBIA |
| XDH |
| TOP1 |
| MDM2 |
| APP |
| MMP1 |
| PCNA |
| ERBB2 |
| PPARG |
| HMOX1 |
| CASP7 |
| ICAM1 |
| MCL1 |
| BIRC5 |
| IL2 |
| CCNB1 |

TYR
IFNG
IL4
TOP2A
GSTP1
XIAP
SLC2A4
INSR
CD40LG
PTGES
NUF2
ADCY2
MET
NOX4
AKR1B1
CDK5R1
MAOA
FLT3
CA2
CCNB3
ALOX5
ADORA1
CA7
GLO1
SYK
GSK3B
PARP1
TTR
CA12
CA4
MMP12
CD38
CYP1B1
ABCG2
AKR1B10
TNKS2
TNKS
ARG1
PTPRS
ABCC1
HSD17B1
ACHE
CDK6
ABCB1

HSD17B2
CYP19A1
ESR2
ADORA2A
CSNK2A1
ALOX15
ALOX12
ESR1
CFTR
AMY1A
GRK6
CA1
CA9
CDK2
TERT
CDK1
AHR
ESRRA
GPR35
AVPR2
IGF1R
F2
PIM1
AURKB
DRD4
MPO
PIK3R1
DAPK1
PYGL
SRC
PTK2
KDR
MMP13
MMP3
CA3
PLK1
CA6
PKN1
CA14
NEK2
CXCR1
CAMK2B
ALK
NEK6

| |
|---|
| PLA2G1B |
| CA5A |
| BACE1 |
| AXL |
| NUAK1 |
| AKR1C2 |
| AKR1C1 |
| AKR1C3 |
| AKR1C4 |
| CA13 |
| AKR1A1 |
| PFKFB3 |
| PLG |
| KDM4E |
| CDK5 |
| CCNB2 |

Supplementary table 2. Target genes corresponding to microglia polarization

| **target name** |
|---|
| ADORA2A |
| AGER |
| AHSG |
| AKT1 |
| ANXA1 |
| ARG1 |
| AXL |
| BIRC3 |
| C3 |
| CCL2 |
| CCL20 |
| CCL3 |
| CCL5 |
| CCR2 |
| CCR5 |
| CD200 |
| CD274 |
| CD4 |
| CEBPA |
| CEBPD |
| CHRFAM7A |
| CNR2 |
| CSF1 |

CSF1R
CXCL10
CXCL12
CXCL8
CYSLTR2
DAB2
DDR1
DOCK2
ELAVL1
EPO
EZH2
F2RL1
FAS
FLT1
FPR2
GRK2
GRN
HAVCR2
HMGB1
HMOX1
HTR2B
ICAM1
IDO1
IFNG
IGFBP2
IKBKB
IL10
IL16
IL1A
IL1B
IL23A
IL4
IL6
IRF9
ITGAM
KCNN4
KDM6B
KIDINS220
LCN2
LGALS3
MALAT1
MERTK
MIR146A
MIR148A

MIR155
MIR21
MIR26B
MIR367
MRC1
MSR1
MTOR
NEWENTRY
NFKB1
NLRP3
NOS2
PDCD1
PELI1
PHB1
PLA2G4A
PPARG
RHOA
RNASET2
ROCK1
ROCK2
SIRT1
SOCS1
SPP1
STAT1
STAT3
TAFA3
TGFB1
TLR2
TLR4
TNFAIP3
TP53
TREM1
TYROBP
VEGFA
WNT5A
CRYAB
CHMP4B
TREM2
VIM
STK11
APP
TNF
MARK2
MAPT

MIP
CX3CR1
RAC1
PTGS2
PRKCI
MSN
APOE
SNCA
CD40
PRKCZ
MAPK14
CXCR4
CCL4
MAPK1
TSPO
RAB8A
BDNF
PIK3CG
SOD1
NUMA1
MAPK8
TJP1
CCR7
CCR3
ABCB1
GFAP
TLR3
MPP1
CTNNB1
EZR
AARS2
SYK
PSEN1
IFNB1
IL18
PRNP
PRKAA2
PRKCA
ABCC1
NOS1
SPI1
TMEM119
MMP9
CXCR3

PTK2B
CD163
MBP
P2RY12
VAV1
TUBB3
SLC1A3
CASP3
P2RX7
PTEN
GSK3B
MMP14
CLASP2
CX3CL1
CSF2
JUN
FGFR1
IDE
VIP
P2RX4
S100B
CCL21
PTK2
CD68
CD36
SLC9A1
ALB
SCARB1
MMP3
PRKAA1
EGFR
NOS3
TLR5
SCN1A
CD40LG
CD33
SOCS3
LOC102724058
CXCR2
PIK3R1
NGF
IL34
FGF2
MAPK10

RDX
CD14
MAPK3
DLG4
GLS
MMP12
IL17A
TUBA1B
RPS27A
CD86
CCL11
IL13
NFKBIA
IRF3
SPN
CAV1
HTT
GDNF
CFH
FASLG
TUBB
MMP2
PIK3CA
SLC1A2
LRRK2
FZD1
IL2
PLAU
PLCG2
CLU
APOA1
CASP8
ERVW-1
IFNA1
CD44
TFRC
ICAM5
ZC3H12A
TLR9
GJB1
CDKN3
VGF
TF
ITIH4

FOS
BMP6
ITGB2
CD80
ICAM3
SRC
IGF1
IRF1
PIK3CB
C5AR1
TIMP1
CYBB
STK4
HSPD1
PTPN11
MMP7
GRIN2B
ABL1
CTSB
CD200R1
TLR7
CCR4
SYP
NTF3
S100A9
CREB1
WNT1
MYH10
PTGER2
PTPRC
TAT
CAT
RELA
CASP1
CCR8
PAK1
MTERF1
HCK
MAP2
STAT6
INPP5D
TGFB2
ANK1
ADCYAP1

PRKD1
PLAUR
IL1R1
JAK2
CD46
EDN1
LAMP1
NOX4
DNAH8
SPHK1
F2
SLC2A5
EP300
TNR
CNR1
CLIC1
IL15
PRKAB1
MAP2K1
HSPG2
AQP4
PTPN6
CSPG4
KLRK1
PXN
CCNT1
ABCC4
HLA-G
CXCL16
NTRK2
CST3
GBA
LCP1
MAPKAPK2
VEGFC
CXCR6
ESR2
F11R
OPRK1
NPC1
DAO
TIMP3
IL12B
F2RL2

MAPK9
PRKN
SERPINE1
IL33
JMJD6
RHO
NR3C1
FGFR2
ARHGEF7
PTGS1
IL12RB2
EPOR
CCL7
OPTN
ITPR1
HGF
TRAP1
LEP
ADCY10
TRAF6
YY1
SERPINF2
BIN1
WARS1
PROM1
ANXA2
NTF4
APOB
SLC8A1
TH
HLA-DRA
FYN
ADAM17
IQGAP1
STAT5B
CDH2
HDAC2
CD74
AIMP1
RELB
IL3
IL1RN
IL27
NFE2L2

ITGAL
CNP
COX5A
IL12RB1
MMP1
NF1
S100A8
IDH1
PPARA
HSP90AA1
OPRM1
CFB
CXCR1
CXCR5
ALOX5
WNT2
NRG1
CIITA
SP1
GPNMB
IGF2
LIF
DNM3
AARS1
PDIA3
SOCS2
MAP1B
PAFAH1B1
MYD88
KCNA3
NR4A2
BRCA1
XDH
ADRB2
TACR1
BECN1
TNFRSF9
CCL1
ESR1
ABCG2
CYCS
CEBPB
CTSL
BTK

LRP1
PVALB
GJA1
CHRNA7
CGAS
TGFA
CNTF
SRR
LPAR2
RAF1
PLS3
GRB2
CXCL1
STAT5A
BCAR1
MST1
CCN2
TGM2
SGCB
HTRA2
RPS6KA1
CTSD
SERPINA3
CD59
CD47
NUDT6
CCR6
F2R
AREG
TYK2
TERT
SAMHD1
HTRA1
OSM
RPS6
ALOX15
LYN
SLC22A2
ERBB2
KRAS
SERPINB2
CRH
PRKCB
NES

INS
FSCN1
TGFBR1
GLG1
INPPL1
ADCY1
MIF
KLK6
AZU1
GALC
MAPK7
GLUL
SOAT1
MAP1LC3A
CDK5
JAK3
ADAM10
PQBP1
CSTB
HSPA5
CHGA
GUSB
IL6R
CRP
MME
ANXA5
LGMN
OLR1
IL18R1
GAP43
AKT1S1
FLT4
PTGER4
LPAR3
HIF1A
SQSTM1
HLA-DRB1
DUSP1
TMSB10
MBL2
EIF2B4
MIRLET7A1
TNFRSF4
TIMP2

BAX
SLC16A1
CD209
NR1H2
TFAM
SIGMAR1
SERPINB3
KCNA2
VCAM1
RPS6KA3
LEPR
PTGDS
OCLN
CASP9
PRKCD
DAGLB
CHIT1
LTF
KITLG
ITGAX
ZAP70
AP2B1
NR3C2
PARP1
HSPA8
LIFR
RAP2A
PSEN2
TBCB
CCR1
BPTF
IL5
EIF2B5
SERPINF1
PDCD10
C1QA
SMAD3
SRF
MPO
ADCY3
ADCY8
TGIF1
LDHA
HSPA4

POLD1
HFE
CD55
CSNK2A1
TMSB4X
CANT1
NGFR
KCND2
VEGFD
CALB1
CD28
CCL17
IL37
SST
EGF
HLA-E
GPR137B
IL4R
PTPN3
TARDBP
C4A
AKT2
SLC6A3
ATP1A3
LPAR1
MELTF
TBK1
PLAT
CCL22
GJC1
ABCA1
PAK4
IRF7
WDR26
ADGRB1
EPHA4
PRDX2
MARCKS
MIR214
GRM5
ERBB4
IGF2R
P2RY2
P2RY6

IL9
CD9
SIRPA
EBAG9
ABCC5
CCL18
TLR8
PTGES
IGF1R
IL6ST
CAPN1
SIK2
TNFSF9
CLDN11
CD69
ITGA6
IL7
RAD51
DKK1
MIR181A1
CREBBP
GSR
TJP2
PLCG1
PKN1
MIR17
PRKACA
FCGR3A
DCD
MIR32
CYFIP1
WASF2
TRAF3
B2M
DUSP19
ABCD1
NCR1
MKI67
P2RY1
FEN1
XIAP
GAS5
NTRK1
ADORA3

PRKCE
MAOB
LHCGR
GCH1
TAC1
TLR1
PIK3C3
LGALS9
TPSAB1
CSNK2B
NRP2
GHRL
HAS2
BRAF
SLC7A1
SRI
AGFG1
HSPB1
CACNA1C
PLXNA1
SERPINA1
ITPR3
ACE
TCF7L2
GPR55
GPX1
PRKCQ
USF1
NFKB2
APOA2
HDAC1
YBX1
PICALM
RTN4
TFEB
CHI3L1
RABGAP1
MDM2
CDKN1A
CCL26
KDM1A
POMC
TPPP
VWF

BCL2
MDH2
DNM1L
PIK3R2
BSG
MAP3K1
FURIN
IL12A
PTGES2
IL2RB
NOD2
CCL8
STEEP1
MAP3K7
NTS
PKM
MIR206
RYR1
DCN
DDX58
PPA1
PHB2
ELANE
BLVRB
MIRLET7B
VTN
ATP6AP2
NTRK3
CSF3
DEFB4A
TAB1
MFGE8
BIRC2
IRAK1
IL2RA
NOX1
TNIK
HCST
HLA-A
IL1RAP
HEXB
GLRX
BCL2L1
EPRS1

ENO1
GAS1
TYRP1
MARCO
MARVELD2
ADCY2
CISH
HEXA
STAT4
FEZ1
ABCG4
CDK9
H2BC21
CLDN5
KCNMB3
GAL
SELPLG
TAX1BP1
IRAK3
SLC8A3
MIR125B1
VDR
S1PR2
APLNR
NTN1
SOD2
NLK
MYCN
IL23R
DAXX
CFL1
EDN2
COP1
MAP2K2
CAVIN1
LDHB
SELL
CTSE
TTF2
FERMT3
IL11
POSTN
CCK
GCK

CFLAR
FGFR3
VSIR
IL22
ITGB3
MX1
OPRD1
NR2F1
CLOCK
PTGES3
HSPA1A
HMMR
DCTN2
PTBP1
RYR2
LRP6
BACE1
QKI
ARNTL
MAP1LC3B
MS4A4A
S100A4
GLUD1
PRDX6
PIK3C2B
FGFR4
NCOA3
XCL1
CBS
IL10RA
BCL2L11
GH1
MIR504
DEFB1
MIRLET7C
TNC
TSC1
ABCB11
PIK3C2A
PLCB1
IKBKG
RAB6A
ARHGDIA
CBX5

MAVS
GLRX2
FTH1
FGF1
PLP1
POLR2A
POLR2E
PURA
CXCL2
ITGA4
RRAGA
EGR1
EDN3
MAPK11
HSPA9
ERGIC2
IL1RL1
RHOH
DCX
SOCS7
CCN4
PDE4A
CASP7
ARMS2
ENPP2
KIF1B
CD22
TIRAP
PLCB3
ACP1
LTB4R
ATP8A1
CHGB
STXBP1
XIST
IGKV2D-29
KIF1A
AKT3
SLC12A5
PIK3CD
CBR1
PTGDR
ATP2A3
MAP2K4

DYNC1H1
MAP4K4
KAT2B
PINK1
HSPA14
PRKACB
BID
PRKCH
HDAC9
PSMF1
RAB1A
LILRB2
MST1R
H19
AKR7A2
IAPP
CORO1A
ADIPOQ
NSF
SMAD7
RBP4
TPI1
POLR2C
SRSF1
PLA2G6
LTA
PGRMC1
FBXW11
NAIP
PDGFRB
EIF2S1
DYNC1I2
RPE
EIF2AK2
SLC30A1
CTLA4
TRPV1
NOTCH1
TNFSF15
IL1RAPL2
CTSG
SELP
F3
PRKD3

ADGRE5
IL21
CGB5
CDKN2A
RB1
RET
MMP8
CD1A
CDK1
PTGER3
HSH2D
PSMB9
HLA-B
CD93
ST2
P2RY4
ITPR2
HEPH
PEBP1
NEU1
SUCNR1
TGFBR2
CTSS
RCAN1
TMED10
PTPA
NPPA
CLEC5A
PTPN1
MAOA
SLC7A11
ADAMTSL1
SCN5A
MIR324
F2RL3
PTGER1
MIR30D
ATRX
KDM6A
HAX1
DYNC1LI2
RAB6B
EPHA3
TRA

F13A1
IRAK4
FGB
TRAF2
NDUFS4
KIF1C
SERPINE2
HYOU1
KIF20A
TP73
TNFSF10
IL32
MIR124-1
NFATC1
MGMT
SMARCA4
CHMP2B
PARL
ABCA3
S1PR1
TLR6
FOXP3
ACHE
RPH3A
P2RX2
HSPA6
CD83
BRD4
TRPC3
RNF2
ADNP
HVCN1
IDUA
RARRES2
IFN1@
P2RY11
CALU
MATR3
MPIG6B
MRGPRX1
LIMK1
GPX4
HLA-C
TK1

TFPI
S100A6
ICAM2
NR2C2
APOD
AGTR1
PSAP
GRIN1
TICAM1
ADA2
PRL
GP2
PF4
TOMM40
LAMP3
RIPK1
SMN1
MIR27A
CCND1
GAK
PPM1B
SLC11A2
PLA2G7
ARSA
ATP2A2
LOX
TKT
ERN1
HSPA2
GPRIN3
PNOC
NCAM1
NCF2
CALR
REL
GRIN2A
MPZ
DDOST
P2RX1
SMN2
CALML5
NCSTN
JAK1
IGFBP3

KCNMA1
FOXO3
WT1
DPEP1
HTR2A
RELN
NR4A1
BIRC5
AKR1A1
C3AR1
ANXA11
PLA2G2A
COLEC12
CDKN1B
PRKCG
IL27RA
BAG3
SNAP25
SUCLA2
ISG15
ASL
CNTFR
S100A11
PTP4A2
POLR2J
POLR2H
POLR2I
KIR2DS2
ACTA2
TNFRSF12A
DBH
CMKLR1
CES1
NINJ1
PPARGC1A
MIR150
ADAMTS13
S1PR3
FTL
CNTNAP2
CP
PTX3
LAMB1
HTR7

ATF3
PCNA
MIR34A
NPY
FGF9
P2RX3
CLEC12A
PLG
CSF3R
S100A12
CD8A
FPR1
BCL11B
NCF1
CYP19A1
IFNGR1
PLCB2
IL13RA1
MRC2
PRKAR2A
IGFBP4
ETS1
ADA
CCNA2
STAR
MIR122
CASP4
SLC16A2
CHUK
LDHD
RCOR1
CALCA
TNFAIP6
FNDC5
CD63
CD34
SPARC
CTRL
GINS2
ATP7A
APOBEC3G
HSF1
RFX1
DYNC1I1

GDF15
C5
KCNH2
PREP
CD300LF
TNFRSF1A
SEMA3A
IFI16
MIR223
CCL13
PIKFYVE
GNPAT
IRS1
SLIT2
C5AR2
NR4A3
GHSR
ADAMTS4
C9orf72
TNFSF12
NEDD4
KEAP1
CYP27B1
FBLN1
APOC2
AGT
GRM2
ULK1
UBQLN1
MARCKSL1
BCHE
H1-1
PLTP
SELE
ABCC8
EPHB2
TNFSF13B
HTR6
NFYA
HPSE
SMAD6
APAF1
DDIT3
HBEGF

TNFRSF6B
ALDH9A1
SESN3
GRIN2C
SEMA7A
SNCB
TRIM37
CRHBP
OSTM1
MIR142
NLRP12
PRKAR2B
LPL
PTGFR
EOMES
CXCL5
SLC18A2
NLRX1
MSI1
NSUN4
STING1
CASR
THBD
ABCG1
SUV39H1
TNFRSF1B
NEFL
INSR
HSPA1B
TRPV4
GLS2
IFITM3
SLC2A12
SCG2
FGF21
CSN3
SGK1
IL7R
BST1
PECAM1
H3C1
FAAH
INHBA
HSPB8

POLR2L
ERP29
DDR2
MAPK12
NEFH
CALB2
ADM
MZF1
GRIA2
MEF2A
PLCB4
OSMR
MLC1
CXCL13
RGS19
MIR133B
COL18A1
MAP3K5
RASGRP1
VTCN1
HCRT
TPPP3
GDA
C1S
SP3
APLP2
TSC22D1
SIGLEC9
ADK
ALOX12
MIR340
MAP2K5
FLT3LG
H4C1
MEF2C
VLDLR
MAG
PADI4
ALDOC
HDAC3
FOLH1
OTC
PRKCSH
FHIT

TWIST1
TOLLIP
KCNH1
A2M
MAP3K13
P2RX6
CHI3L2
ASS1
ENO2
NAGLU
ABCA2
STK16
LAMB2
NR1I3
GRM1
ADORA1
E2F1
VEGFB
JUND
SUCLG2
ILRUN
PCSK9
UGT1A1
PLCD1
PPT1
IFNGR2
PROX1
S100A13
PDGFRA
GSTM3
OGDH
SLC7A5
NID1
CRABP2
PLA2G10
METTL3
RUNX1
CPA6
NPY4R
GGA1
SBNO2
PDE4D
SMPD1
GRIA1

TCF4
GHR
C1QC
C1QB
PRDM1
PRDX5
CYSLTR1
THY1
IL15RA
DLX2
PACSIN3
ARSH
FST
KCNK9
SSTR2
SERPING1
IFNAR1
ABCC9
MSTN
DLL4
DRD3
CAMKK1
MAN2B1
TPP1
GCKR
P2RY14
SEL1L
CSNK1G1
IGFBP1
HLA-DPA1
IL21R
POLR2D
DIO3
LTB
HIVEP2
HRK
FAM3B
NLRP9
ELOF1
TPRA1
SPPL2C
AKIRIN2
RIPOR2
RIPOR1

Supplementary table 3. Cross-targeting of drugs and diseases

| **Common Target** |
|---|
| MMP2 |
| XDH |
| ALOX5 |
| BIRC5 |
| MAOA |
| CDK1 |
| PTK2 |
| ADORA2A |
| PTGES |
| PPARG |
| ALOX15 |
| ABCB1 |
| CYP19A1 |
| TNF |
| MPO |
| PCNA |
| F2 |
| XIAP |
| PTGS2 |
| ARG1 |
| NOX4 |
| CCND1 |
| ESR1 |
| CASP7 |
| VEGFA |
| ACHE |
| GSK3B |
| MMP1 |
| IL6 |
| MMP12 |
| CASP3 |
| PARP1 |
| ABCG2 |
| HMOX1 |
| ALOX12 |
| MMP3 |
| AXL |
| CXCR1 |
| BACE1 |
| IL10 |
| MAPK1 |
| PIK3CG |

MDM2
EGFR
ADORA1
IL2
ERBB2
SYK
IFNG
IL4
IGF1R
ICAM1
BCL2L1
TERT
RELA
RB1
SRC
PKN1
PLG
TP53
INSR
CASP9
CDK5
CDKN1A
AKT1
PIK3R1
ADCY2
ABCC1
NFKBIA
PTGS1
AKR1A1
JUN
ESR2
CSNK2A1
CD40LG
APP
MMP9
